# Inhibition of Mitochondrial Complex III Blocks Neuronal Differentiation and Maintains Embryonic Stem Cell Pluripotency

**DOI:** 10.1371/journal.pone.0082095

**Published:** 2013-12-02

**Authors:** Sandro L. Pereira, Mário Grãos, Ana Sofia Rodrigues, Sandra I. Anjo, Rui A. Carvalho, Paulo J. Oliveira, Ernest Arenas, João Ramalho-Santos

**Affiliations:** 1 CNC- Center for Neuroscience and Cell Biology, University of Coimbra, Coimbra, Portugal; 2 Department of Life Sciences, Faculty of Sciences and Technology, University of Coimbra, Coimbra, Portugal; 3 Biocant – Technology Transfer Association, Cantanhede, Portugal; 4 Laboratory of Molecular Neurobiology, Department of Medical Biochemistry and Biophysics, Karolinska Institute, Stockholm, Sweden; University Hospital of Münster, Germany

## Abstract

The mitochondrion is emerging as a key organelle in stem cell biology, acting as a regulator of stem cell pluripotency and differentiation. In this study we sought to understand the effect of mitochondrial complex III inhibition during neuronal differentiation of mouse embryonic stem cells. When exposed to antimycin A, a specific complex III inhibitor, embryonic stem cells failed to differentiate into dopaminergic neurons, maintaining high Oct4 levels even when subjected to a specific differentiation protocol. Mitochondrial inhibition affected distinct populations of cells present in culture, inducing cell loss in differentiated cells, but not inducing apoptosis in mouse embryonic stem cells. A reduction in overall proliferation rate was observed, corresponding to a slight arrest in S phase. Moreover, antimycin A treatment induced a consistent increase in HIF-1α protein levels. The present work demonstrates that mitochondrial metabolism is critical for neuronal differentiation and emphasizes that modulation of mitochondrial functions through pharmacological approaches can be useful in the context of controlling stem cell maintenance/differentiation.

## Introduction

Although mitochondrial involvement in stem cell biology is far from being completely understood, the possible use of mitochondrial modulation to improve stem cell culture, differentiation and, more recently, reprogramming, has raised interest in recent years [1-6]. Embryonic stem cells (ESCs) and induced pluripotent stem cells (iPSCs) are characterized by unlimited self-renewal and pluripotency. ESCs are derived from the inner cell mass (ICM) of the pre-implantation blastocyst [7,8], the former physiologically existing in a relatively hypoxic environment (1.5-5.3% O_2_) [9]. Accordingly, ESCs present a prevalent glycolytic metabolism and human ESC have been shown to be better maintained under hypoxic culture conditions [10,11]. Interestingly these cells are capable of robustly growing under normoxia, while maintaining the same metabolic pattern [11,12]. To complete reprogramming, iPSCs embrace a metabolic shift from aerobic oxidative phosphorylation (OXPHOS) present in the initial differentiated state, towards glycolysis, thereby acquiring a metabolic energy profile that is comparable to ESCs [13-16]. Indeed this metabolic shift precedes the onset of endogenous pluripotency marker expression [17]. Furthermore, hypoxic conditions favor the reprograming process, both for mouse and human cells [18]. 

Aerobic glycolysis is a recurrent metabolic pattern in rapidly proliferating cells, including cancer cells, first described by Otto Warburg in what is now known as the Warburg effect [19]. Despite apparently representing a less efficient metabolic process than aerobic mitochondrial OXPHOS, glycolysis endows rapidly proliferating cells with several advantages: a) fast ATP generation; b) decreased mitochondrial oxidative stress, as a consequence of reduced reactive oxygen species (ROS) generation in mitochondria, and increased NADPH formation, a substrate for antioxidant defenses regeneration in the pentose phosphate pathway; c) fast production of precursor compounds used for the synthesis of biomolecules [20-22].

The metabolic architecture of ESCs resembles what takes place in early development, particularly concerning mitochondria. Throughout initial embryo cleavage a reported bottleneck effect restrains mitochondrial DNA (mtDNA) replication and mitochondrial biogenesis, resulting in a drastic reduction in mitochondrial mass per ICM cell [4]. Furthermore, mitochondria in ICM cells are small organelles with translucent matrix and few cristae, which is typical of an immature morphology [4]. Both ESC and iPSC are reported to share these mitochondrial properties [13-15,23-25].

Contrarily to cell reprogramming, conversion of pluripotent stem cells (whether ESCs or iPSCs) into differentiated phenotypes involves a glycolytic to oxidative metabolic transition, accompanied by a coordinated genetic and metabolic restructuring. This is especially evident if the resulting cells have high ATP requirements, such as neurons [26-29]. Although some contradictory results have been reported [30], the emerging trend assumes that ESC differentiation involves an increment in mitochondrial mass, with a concomitant increase in more mature mitochondrial morphology [24,25,28]. This increased mitochondrial mass is accompanied by a rise in O_2_ consumption and ATP production, as well as a decrease in lactate production. Furthermore, mtDNA or nuclear mutations affecting mitochondrial proteins precluded the completion of cell differentiation [31]. 

Mitochondrial remodeling during pluripotent stem cell self-renewal, differentiation and reprogramming, suggests that modulation of mitochondrial functions may serve as a tool to control both processes. In fact, treatment of both human ESCs (hESCs) and mouse ESC (mESCs) with mitochondrial complex III inhibitors antimycin A (AA) or myxothiazol, or mitochondrial membrane potential (MMP) uncoupler such as Carbonyl Cyanide m-Chlorophenylhydrazone (CCCP), increases the expression of pluripotency markers and enhances cell pluripotency [32,33], inhibiting spontaneous stem cell differentiation [32]. 

Information on the effects of mitochondrial modulation during the differentiation of stem cells into neurons is scarce. A suggestive work of Vayssiére and colleagues using clonal cell lines with neuroblastoma origin showed that MMP uncoupling, the inhibition of mitochondrial translation and the inhibition of DNA, RNA and protein synthesis, all had a negative impact on cell maturation [34]. Interestingly the observed effect did not seem to result from the cells being energetically compromised, thus suggesting alternative mitochondrial functions in the differentiation process. 

AA is an established chemical inhibitor of the electron transport chain complex III, known to act by inhibiting electron transfer from the Qp to the Qn site of that complex, resulting in the accumulation of the semiquinone radical, thus also enhancing the possibility for reactive oxygen species formation [35]. The objective of the present work was to evaluate if inhibition of mitochondrial functions by AA limits specific differentiation of mESC into neurons by maintaining the pluripotency state of the former.

## Material and Methods

### Cell culture and differentiation procedure

Mouse embryonic stem cell lines E14Tg2a and R1 were kindly provided by Dr. Domingos Henrique (Instituto de Medicina Molecular, Lisbon, Portugal) and originally referenced in [36], and Dr. Andras Nagy (Mount Sinai Hospital and Samuel Lunenfeld Res. Inst., Toronto, Canada), originally referenced in [37] respectively. Cells were maintained and propagated in 0.1% gelatin treated plates in ES-Serum Replacement Media (SRM), composed of KnockOut-DMEM (#10829-018; Life Technologies), 15% KnockOut serum replacement (#10828-028; Life Technologies), 1% non-essential amino acids (#11140-035; Life Technologies), 0.1 mM mercaptoethanol (#M-7522, Sigma-Aldrich), 200 mM L-glutamine (#25030-024; Life Technologies) and 100 U/ml penicillin/streptomycin (#15140-122; Life Technologies), with Leukemia inhibitory factor (LIF) (#ESG1107; Chemicon - Millipore) supplementation.

Dopaminergic (DA) neuron differentiation was accomplished by co-culturing mouse embryonic stem cells (mESCs) with PA6 stromal cells according to previously described protocols [38,39], with adaptations. Briefly, mESC (R1 and E14 cell lines) were seeded (65 cells/cm^2^) on mitomycin-treated PA6 cells and cultured in SRM without LIF. After 5 days, the medium was changed and supplemented with 200 ng/ml Shh (#461-SH; R&D Systems) and 25 ng/ml FGF8 (#423-F8; R&D Systems). From day 8 to day 12 cells were cultured in N2 medium consisting of a F12 (#21765-029; Life Technologies) and MEM (#21090-022; Life Technologies) mixture 1:1, N2 supplement (#17502-048; Life Technologies), 15 mM HEPES, 1mM glutamine, 3 mg/ml ALBUMAX (#11020-021; Life Technologies) supplemented with 250 ng/ml sonic hedgehog (Shh), 25 ng/ml fibroblast growth factor 8 (FGF8) and 20 ng/ml basic FGF (bFGF) (#233-FB; R&D Systems). On day 11 the medium was replaced with N2 medium supplemented with 30 ng/ml brain-derived neurotrophic factor (BDNF) (#248-BD; R&D Systems), 30 ng/ml glial-derived neurotrophic factor (GDNF) (#212-GD; R&D Systems), and 200 mM ascorbic acid (#A-7506; Sigma-Aldrich). The mitochondrial inhibitor antimycin A (50 nM, #A8674; Sigma-Aldrich) was added to the cultures at days 2, 3.5, 5, 6.5, 8, 9.5, 11 and 12.5 following media replacement. Monolayer differentiations were conducted as described elsewhere [40,41]. mESCs were seeded in gelatin-coated plates at a density of 10,000 cell/cm^2^ in ES-SRM. 24 hours later, the medium was changed to a N2/B27 mixture. Medium was renewed at days 5 and 8 of differentiation or preceding every antimycin/vehicle (ethanol) treatment. Areas from the colonies were evaluated by processing optical microscopy photographs using the open-source image analysis software ImageJ.

### Immunocytochemistry/High resolution ELISA

At the end of the differentiation protocols (day 14) or at the referred time points (see Results) cells were fixed with 4% paraformaldehyde (PFA) and submitted to immunocytochemistry (ICC) or high-resolution ELISA, to monitor the presence of Oct4 (pluripotency marker), βIII-tubulin (TuJ1, neuronal marker) and tyrosine hydroxylase (TH, dopaminergic neuron marker). Briefly, cells were blocked in PBS containing 3% bovine serum albumin (BSA) and 0.25% Triton X-100 for one hour at room temperature (RT) and stained overnight at 4 °C with the following primary antibodies: mouse monoclonal anti-βIII-tubulin (1:500, #G712A; Promega), rabbit polyclonal anti-tyrosine hydroxylase (1:500, #P40101-0; Pel-Freeze) and rabbit monoclonal anti-Oct4a (1:200, #C30A3; Cell Signaling). Secondary antibodies were incubated for one hour at RT. For ICC FITC-AffiniPure goat anti-mouse IgG (1:500, #115-095-146; Jackson Immunoresearch) and Texas Red-X goat anti-rabbit IgG (1:200, #T6391; Molecular Probes) were used, and nuclei counterstained with Hoechst 33342 (#H1399; Molecular Probes) 

The numbers of total and TuJ1 and TH immunoreactive colonies were manually counted based on the presence of integral neural structures, using duplicates for each experimental condition within each experiment. High resolution ELISA was adapted from a protocol described elsewhere [42]. The procedure was similar to the one described above for ICC with some modifications. Prior to the blocking step, cells were permeabilized with 0.1 % Triton X-100 in PBS and endogenous peroxidase activity was quenched by treatment with 3% H_2_O_2_ solution in PBS for 5 minutes. Secondary antibodies used were conjugated to horseradish peroxidase (HRP): anti-rabbit IgG (1:500, #7074; Cell Signaling) and HRP linked anti-mouse IgG (1:500, #7076; Cell Signaling). HRP activity was detected by incubation with the chromogenic substrate tetramethylbenzidine (1-step Ultra TMB-ELISA, Thermo Scientific) and the reaction was stopped with 1 M H_2_SO_4_ after a 10 to 15 minute incubation depending on the protein to be detected. Signal quantitation was obtained by determining the optical density at 450 nm in a Microplate Spectrophotometer PowerWave XS, (BioTeK). To define the target protein relative concentration optical density values were first normalized by total cell mass (as assessed by the sulforhodamine B assay, see below) and then to the respective control. 

Relative total cell mass quantification using sulforhodamine B (SRB) (#S1402; Sigma-Aldrich) was performed with some adjustments to the protocol previously reported [43]. After HRP reaction cells were thoroughly washed with PBS and dried at room temperature (RT). Cells were then stained with 0.5% (w/v) SRB in 1% acetic acid for 1 h at 37 °C. Unbound dye was removed with 1% acetic acid. SRB bound to cell proteins was extracted with 10 mM Tris base solution, pH 10, and the optical density was determined at 450 nm. At least 3 independent experiments were performed for each cell line.

### Flow Cytometry

Proliferation of ESCs was evaluated by propidium iodide (PI) and bromodeoxyuridine (BrdU) incorporation followed by flow cytometry analysis. R1 and E14 cells were plated on 6 well plates (10,000 cells/cm^2^) and cultured for 4 days in the presence of AA (50 nM). Medium and treatments were renewed daily. Cells were incubated with BrdU (50 μM, #B9285; Sigma-Aldrich) for 1 hour, thoroughly washed with PBS, detached, fixed with ethanol (70%) and kept overnight at -20°C. Alternatively, after BrdU incubation, cells were washed and cultured for 4 additional hours prior to the fixation procedure. Staining protocol was preceded by DNA acidic denaturation with HCl (2N) for 15 minutes at room temperature. Both anti-BrdU mouse monoclonal primary antibody (1:250, #B2531; Sigma Aldrich) and goat anti-mouse FITC secondary antibody (1:300, #115-095-146; Jackson ImmunoResearch) were sequentially incubated for 30 minutes at room temperature in the dark. Samples were treated with PI/RNase staining buffer (#550825; BD Pharmingen) for 15 minutes at room temperature and subsequently analyzed on a Becton Dickinson FACSCalibur cytometer, using FL1 and FL3 channels for BrdU and PI detection respectively. For the neural precursor marker nestin, fixed cells were blocked in PBT (0.5% BSA + 0.1% Tween 20 in PBS), incubated with anti-nestin mouse monoclonal antibody (1:50, #sc-58813; Santa Cruz Biotechnology) followed by staining with the previously referred secondary goat anti-mouse antibody. Data acquisition and analysis was performed in CellQuest Software (BD Biosciences). PI histogram modeling was performed in ModFit LT software (Verity Software House). 

### Caspase-3/7 activity

Possible pro-apoptotic effects of AA in mESC were studied by the use of the Caspase-glo 3/7 assay (G8091; Promega). Cells were seeded in 24 well plates at a density of 10,000 cells/cm^2^. AA treatment was initiated on day 0 and reapplied every day with simultaneous medium renewal. At day 4, cells were detached by accutase (#A11105-01; Life Technologies) treatment, counted and a total of 20,000 cells per condition were used. This cell number normalization was adopted to exclude possible proliferation effects what would noticeably affect total cell numbers at day 4. Cells were centrifuged (200xg for 5 minutes) and ressuspended in 100 µL of mESC proliferation medium. Equal volume of the caspase-glo 3/7 reagent was added, and samples were transferred to a white walled 96 well plate (Corning). After a gentle homogenization followed by an incubation of approximately 2 hours at room temperature, the plate was read in a LUMIstar Galaxy luminometer (BMG LABTECH).

As a positive control, cells cultured for 4 days in control conditions were incubated with H_2_O_2_ (0.5 mM) and caspase 3/7 activity was evaluated 4 hours later. 

### Immunoblotting Detection of HIF-1 alpha

Total cell extracts were obtained by scrapping cells with heated 2x Laemmli buffer (supplemented with DTT 350 mM) after medium aspiration and rinsing the cells in ice-cold PBS. Buffer volume was normalized by total cell number (100 µL per 1x10^6^ cells) determined on replicate wells. Extracts were immediately heated at 95°C for 5 minutes, submitted to 3 freeze-thaw cycles in liquid nitrogen and then kept at -80°C or immediately processed. Subsequent to an additional denaturing step (95°C for 5 min), equal volumes of extract samples were electrophoretically separated on a 7.5 % SDS-polyacrylamide gel. Proteins were transferred to polyvinylidene fluoride (PVDF) membranes (Trans-Blot® Turbo™ Transfer Packs, Bio-Rad) using a Trans-Blot^®^ Turbo™ Transfer System (transfer program: 30 min at 25 V constant). Membranes were blocked with 5% skim milk powder in PBS-Tween 20 (PBS-T) [0.1% (v/v)] and then incubated at RT for 2 hours with rabbit anti-HIF-1α (1:1,000, #PA1-16601; Thermo Scientific) and mouse anti-actin (1:10,000, #MAB1501; Millipore) primary antibodies. Secondary antibodies conjugated with alkaline phosphatase: goat anti-mouse (1:10,000, #155-055-003; Jackson ImmunoResearch) and goat anti-rabbit (1:3,000, #111-055-003; Jackson ImmunoResearch) were incubated for 1 hour at RT. Protein-immunoreactive bands were developed using the Enhanced Chemifluorescence (ECF) detection system (GE Healthcare) and visualized in a Molecular Imager FX System (Bio-Rad). Immunoblot results were analysed by calculation of adjusted volumes (total intensities in a given area with local background subtraction) for each immunoreactive band using Quantity One^®^ software version 4.6 (Bio-Rad). All bands were adjusted to the loading control (actin), and normalized to the corresponding control.

### Adenylate Energy Charge

mESCs were treated for 4 days and intracellular adenine nucleotides (ATP, ADP, and AMP) were quantified as described elsewhere [44]. Briefly, cells extracts were performed with 0.6 M perchloric acid supplemented with 25 mM EDTA-Na. After centrifugation, cell supernatants were neutralized with 3 M KOH in 1.5 M Tris followed by a new centrifugation step. Supernatants were assayed by separation in a reverse-phase HPLC using a Beckman-System Gold. The detection wavelength was 254 nm, and the column used was a LiChrospher 100 RP-18 (5 μM, Merck). Adenylate energy charge was calculated according to the following formula: ([ATP] + 0.5 × [ADP])/([ATP] + [ADP] + [AMP]). 

### Detection of Reactive Oxygen Species Production

Superoxide anion production was measured by incubating the cells with the fluorescent probe MitoSOX^TM^ Red (#M36008, Life Technologies). mESC were treated with 50 nM or 50 µM (positive control) of AA, for a total of two hours. After the first one and half hours of the treatment, cell medium was exchanged for MitoSOX^TM^ Red (3.5 µM)-containing HBSS (#14065-049, Life Technologies) supplemented with 5 mM of D-glucose, to which new AA was added. Following an incubation period of 30 minutes at 37°C, cells were washed, incubated with HBSS and observed under a fluorescence microscope. Pictures were taken and fluorescence intensity was evaluated through the ImageJ software. We have determined the corrected total cell fluorescence (CTCF) through the formula (CTCF = Integrated Density – Area of Cell × Background Fluorescence) as reported elsewhere [45]. Several pictures were taken from each condition and all cells from each picture were analyzed.

### Statistical Analysis

Statistical analysis was performed by using SPSS Statistics 17.0 (SPSS Inc.). Assumptions of normality and homoscedasticity were tested and appropriate non-parametric or parametric tests were performed depending on the experimental design, as reported in the legend for each figure. Data are expressed as means ± SEM for the number of experiments indicated. Significance was considered when p ≤ 0.05.

## Results

### Antimycin A treatment inhibits neuronal differentiation

We have previously demonstrated that AA maintains human embryonic stem cell pluripotency under proliferative culture conditions [33]. Our goal here was to investigate a putative AA effect in a cell differentiation context. Therefore, mESCs (E14 and R1 cell lines) were specifically differentiated into neurons with the PA6 co-culture differentiation system, consistently used for the enrichment of midbrain dopaminergic neurons [39]. 

**Figure 1:**
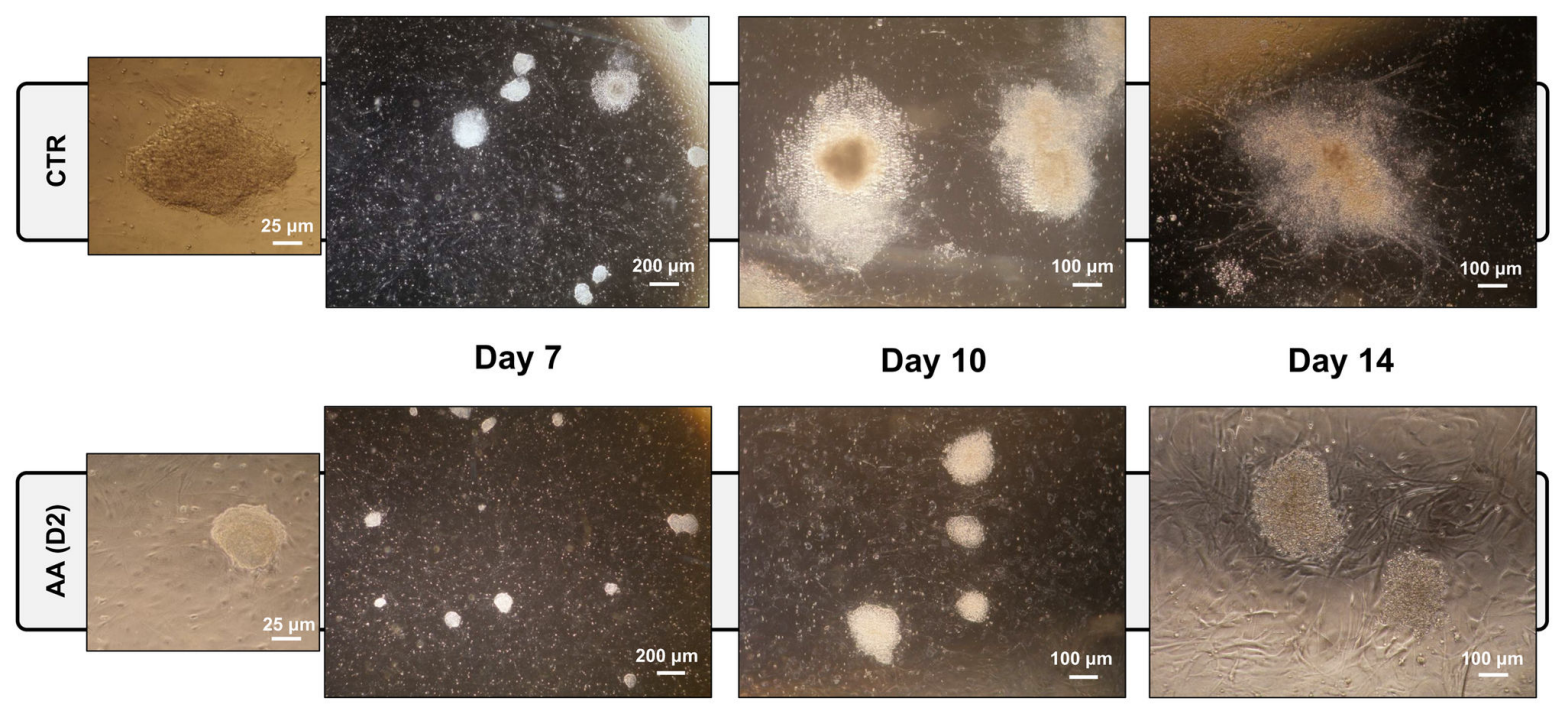
Antimycin A disturbs mESC neuronal differentiation. mESCs differentiated for 14 days into dopaminergic neurons using a PA6-based system were treated with AA since day 2. Representative phase contrast microscopy images were obtained at different time-points of the process (days 7, 10 and 14). AA-treated colonies were smaller and with a more compact morphology resembling undifferentiated mES cells colonies. AA-treatment also led to the absence of neuronal processes, which are easily depicted in control (CTR) colonies at later stages.

AA treatment initiated at day 2 of the differentiation process clearly decreases differentiation efficacy (Figure 1), as AA-treated cells maintain a more compact morphology resembling undifferentiated colonies until later stages of the differentiation protocol. Colony size was equally affected as AA-treated cultures exhibited reduced colony size. Furthermore, neural processes, which were present in the final stages in control (CTR) differentiations, were significantly fewer, smaller and with a lower degree of structural complexity in AA-treated cells (Figure 1 and Figure S1). These observations suggest that AA, when added at day 2 of ESC differentiation into neurons, inhibits or delays the process.

**Figure 2:**
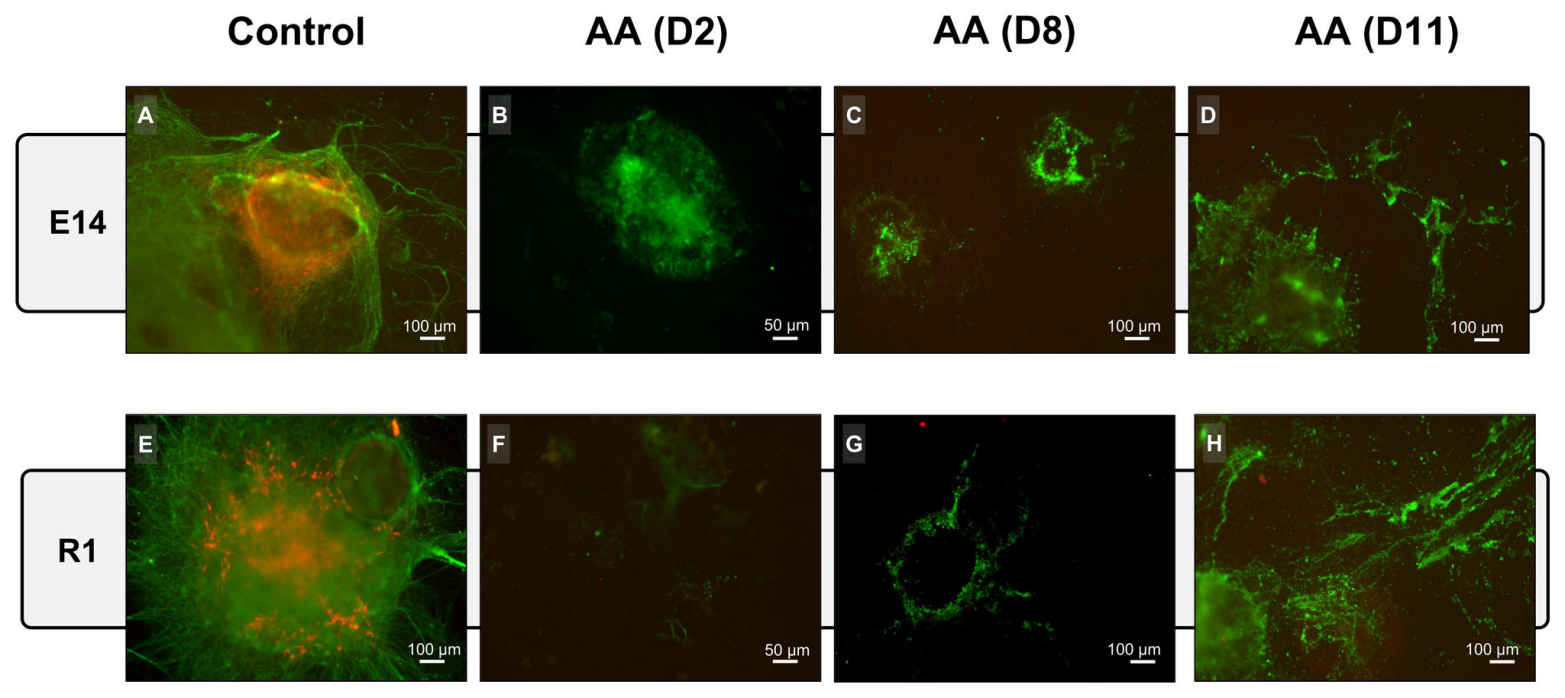
AA affects the mESC neuronal differentiation as seen by TuJ1 and TH labelling. At day 14 of differentiation, cells were stained for the pan neuronal marker Tuj1 (green) and the dopaminergic neuron marker TH (red) by ICC. AA treatment seems to cause stage specific effects on differentiating cell populations. Control conditions show large colonies with complex neuronal processes and consistent numbers of TH-positive cells (A and E). Cells treated with AA starting on day 2 presented colonies that were only positive for Tuj1 but with no complex neurite formations (B and F). AA treatment at later stages resulted in increased cell loss as observed by degraded colonies and loss of neuronal processes (C, D, G and H). The images are representative of at least 3 independent experiments.

**Figure 3:**
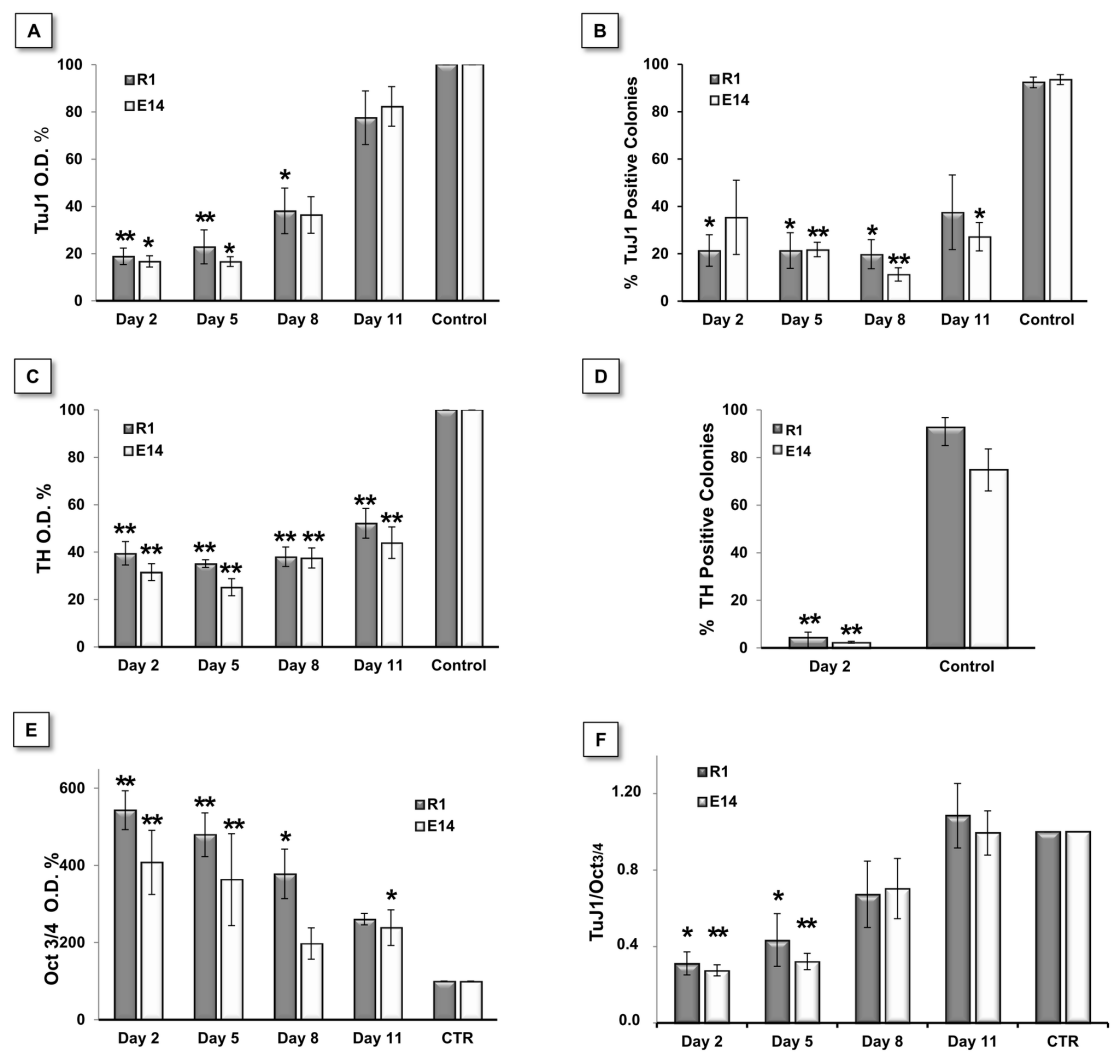
AA quantitatively affects mESC neuronal differentiation and maintains pluripotency. A, B, C and D - Quantification of TuJ1 and TH expression using high-resolution ELISA and manual colony counting on R1 and E14 cells differentiated for 14 days. TuJ1 expression consistently decreased in cells treated with AA starting on day 2, 5 and 8 on both cell lines (A). Significant differences in TH expression exist when comparing all treatment time-points with the control condition (C). Both Tuj1 and TH differences were validated by manual colony counting (B and D). Statistical analysis on ELISA-based data was performed on raw values using a One-way Anova, followed by a Dunnett test. To maintain parametric assumptions TH data was log-transformed. For colony counting data we used a One-way Anova with a Games-Howell test (homoscedasticity not verified) for TuJ1, and Student’s T test for TH. ELISA-based detection of Oct4 pluripotency marker expression showed a clear correlation between Oct3/4 levels maintenance and AA treatments, with the former found increased as treatment with AA was performed in earlier time points (E). Significant differences were found for all groups when compared to control condition except for Day 8 in E14 cells. Raw data was normalized to cell mass (SRB) and then to control levels (CTR=100%). Statistical analysis performed on O.D. values normalized by protein mass (SRB). In order to verify parametric assumptions, R1 data were log-transformed. Data were analyzed by One-way Anova with a Dunnett test. (F) The TuJ1/Oct3/4 ratio was used as a measure of differentiation efficiency. This ratio showed that when added at days 2 and 5, AA significantly decreased differentiation efficiency. Statistical analysis was performed using a One-way Anova, followed by a Tukey post hoc test. Statistical significance considered when p≤ 0.05., (* p<0.05; ** p<0.01). Error bars = SEM (calculated through error propagation formula for D). At least 3 independent experiments were performed.

We further evaluated the expression of the pan-neuronal marker class III β-Tubulin (TuJ1) and the dopaminergic neuron marker tyrosine hydroxylase (TH) at the end of the differentiation protocol (day 14). For this purpose, AA treatment was initiated at different time-points (days 2, 8 and 11), resulting in distinct immunoreactivity against the two markers. As shown in Figure 2, the control group showed large colonies with intricate and complex neuronal processes, which were positive for TuJ1 (green in Figure 2A, 2E). Treatment with AA starting at day 2 (Figure 2B, 2F) resulted in smaller colonies with low TuJ1 labeling in cells with immature neuronal morphology. Moreover, clear TH labeling was absent in the majority of these colonies, while differentiations in the control group displayed a large numbers of cells expressing this marker. Furthermore, colonies in which AA treatment was only initiated at day 8 or 11 (Fig, 2C, 2D, 2G and 2H) revealed a disrupted morphology, with empty areas in the colonies suggestive of cell loss that could be confirmed by the increased presence of cell debris in the culture medium. Nevertheless, these colonies still present residual TuJ1 reactivity. A more objective quantification of marker expression was obtained through high resolution ELISA and manual counting of positive colonies (Figure 3). ELISA quantification showed that the expression of both Tuj1 and TH was high in control condition and decreased with in the presence of AA in both cell lines, in a manner that was inversely correlated with the time at which treatment was initiated (Figure 3A, 3C). Analysis of the number of positive colonies yielded similar results, i.e., a decrease in the number of Tuj1- and TH-positive colonies after AA treatment (Figure 3B, 3D). Although the results obtained by the ELISA assay showed residual TH expression in AA-treated cells (likely due to the lower signal to noise ratio when using this marker), our immunohistochemistry-based manual counting suggest that these were background levels, as the percentage of TH positive colonies was less than 5% (Figure 3C, 3D). 

The presence of the pluripotency marker Oct3/4 was also evaluated by ELISA. As shown in Figure 3E, Oct3/4 expression was significantly higher in cells that were treated with AA earlier in the differentiation process, confirming that the inhibitor contributed to maintain the presence of this pluripotency marker in the culture, despite the triggering of differentiation. This was further supported by the ratio between the percentage of TuJ1 and Oct3/4-positive cells (Figure 3F), a good indicator of differentiation efficiency in this system, even though non-neuronal cells might also be present in culture. Figure 3F clearly demonstrates an inverse correlation between the time at which the inhibitor treatment was performed and the efficiency of the differentiation process, with differentiation after AA introduction at day 2 as the lowest, while drug treatment at day 11 was similar to control cultures.

In order to rule out an indirect effect of AA on PA6 cells, mESC lines were also differentiated without the presence of feeder cells. Despite this being a less efficient process, the protocol was capable of generating a significant number of neurons. Immunocytochemistry for TuJ1 and TH revealed that AA treatment also hindered neuronal differentiation under these conditions, thus reinforcing the idea that this drug prevents the neural differentiation of mESCs (Figure S2). This experiment also stressed that the inhibitory effect is not limited to the dopaminergic lineage, as the number of non-dopaminergic TUJ1^+^ cells were also reduced. 

**Figure 4:**
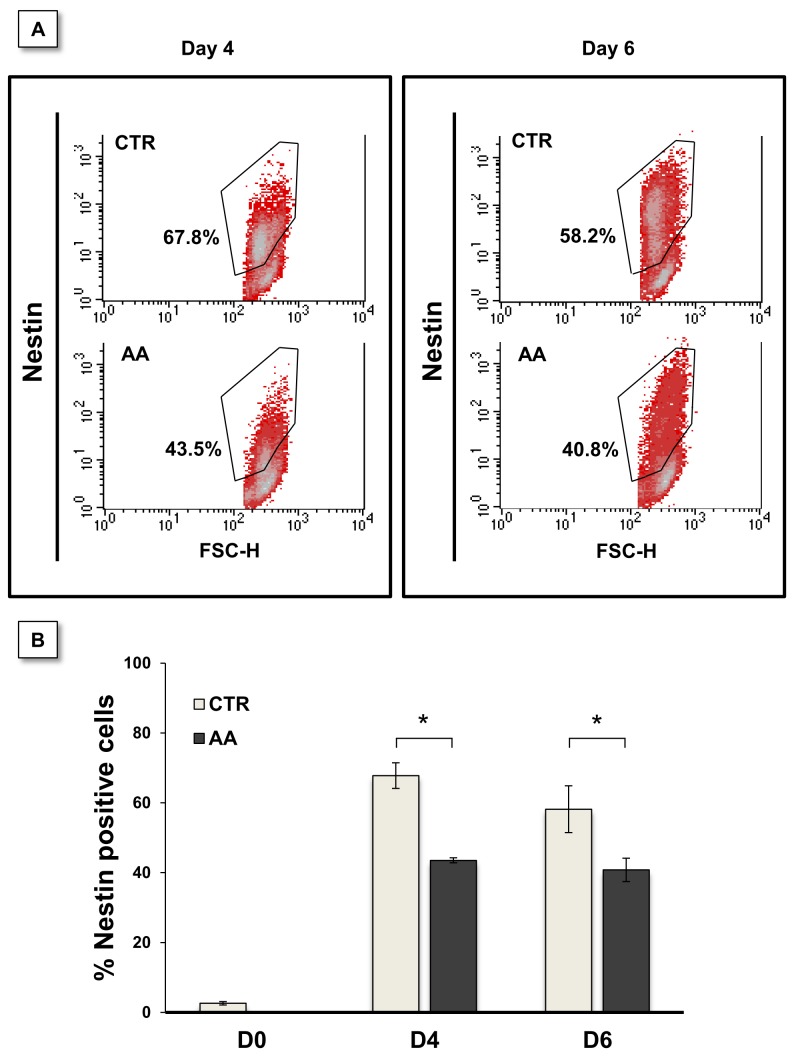
AA treatment causes a reduction in nestin-positive cells. E14 cells were differentiated in monolayers for 4 or 6 days in the presence of AA since day 2. Cells were analyzed by cytometry for nestin labeling (A). Nestin positive cells were reduced on treated cultures when evaluated both at day 4 and day 6. Statistically significant differences were found when comparing control and treated cultures for each time-point (B). Data were analyzed by a two-way Anova for independent samples. Error bars = SEM. Statistical significance considered when *p≤ 0.05. Results express at least 3 independent experiments.

We next assessed the effect of AA at an earlier stage of differentiation, particularly evaluating whether AA had any earlier discernible effect on the differentiation of mESC into nestin-positive neural stem cells. As shown in Figure 4, AA treatment starting on day 2 significantly reduced the numbers of nestin-positive cells, as assessed by flow cytometry. These results were observed both at days 4 and 6 using the monolayer differentiation culture system (CTR = 67.8 ± 3.7 % vs. AA = 43.5 ± 0.7 % at day 4 and CTR = 58.2 ± 6.7 % vs AA = 40.8 ± 3.3 % at day 6), confirming that AA prevents the differentiation of mES cells affecting thus their differentiation into neural stem/progenitor cells, and their subsequent neuronal differentiation..

### Antimycin A affects the proliferation of mouse embryonic stem cells, but does not induce apoptosis

**Figure 5:**
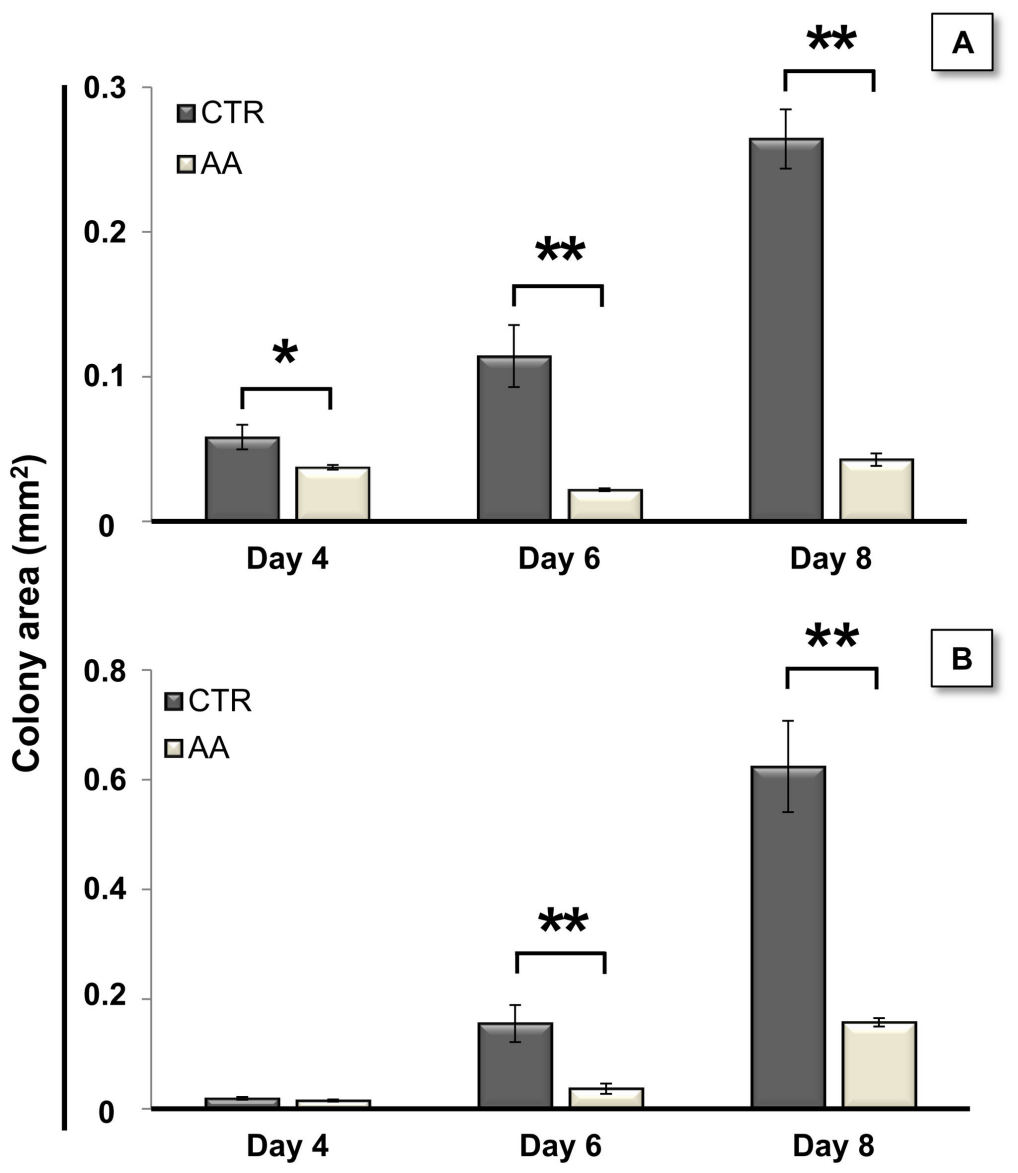
AA reduces colony area. Antimycin A treatments were initiated at day 2 of differentiation using both R1 (A) and E14 cells (B), and repeated every one and half days. Cells were fixed at days 4, 6 and 8 and colony areas were evaluated by processing phase-contrast microscopy photographs with Image J. For both cell lines AA treatment severely decreased the colony area. To verify parametric assumptions by Shapiro-Wilk and Levene tests, area values were submitted to a logarithm transformation. Data were then evaluated by a two-way Anova for independent samples, which revealed a significant interaction between the effects of treatment and time. Subsequent simple main effects analysis showed that the areas from treated and control colonies were significantly different at days 4 (P= 0.03), 6 (P= 0.000001) and 8 (P= 0.000002) for R1 cells and at days 6 (P= 0.0001) and 8 (P= 0.0002) for E14 cells. Error bars = SEM (* p<0.05; ** p<0.01). For better comprehension raw area values from 3 independent experiments are represented.

As described above, AA treatment appears to affect mESC colony size. To confirm this, we differentiated mESC in co-culture with PA6 cells and evaluated the size of the resulting colonies at days 4, 6 and 8. Figure 5 shows that early AA treatment significantly prevents the increase in colony size during differentiation. When initiated at day 2, AA treatment resulted in significantly smaller colonies, starting at day 4 for R1 cells and at day 6 for E14 cells, with clear differences between untreated and AA-treated cells at day 8 of differentiation for both cell lines.

**Figure 6:**
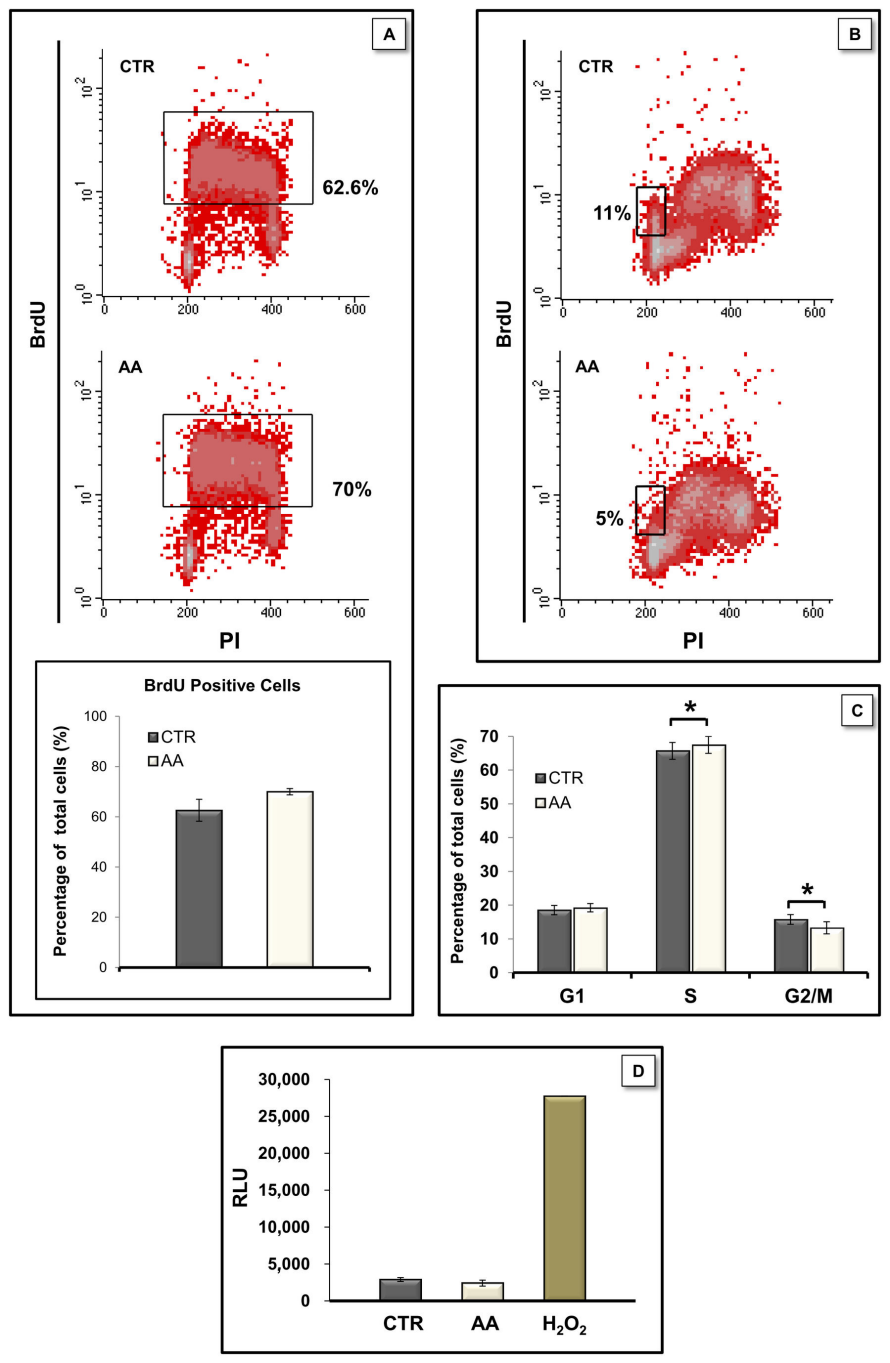
AA affected proliferation, cell cycle analysis but did not induce apoptosis in mESC. E14 cells were cultured for 4 days in proliferation medium in the absence or presence of AA. Cells were then pulsed with BrdU for one hour and immediately fixed (A) or further cultured for 4 more hours in the absence of BrdU prior to fixation (B). Cell cycle analysis by PI staining with flow cytometry (C). Caspase-3/7 activity was evaluated by Caspase-glo-3/7 assay (D) using H_2_O_2_ (0.5 mM) treatment for 4 hours as a positive control. Values are expressed as relative luminescence units and no significant differences were found between caspase activity in CTR and AA-treated conditions (p = 0.197). Data are normal and homoscedastic and were analyzed by paired sample Student’s T tests. Error bars = SEM. Statistical significance considered when p≤ 0.05 (* p<0.05). Results represent at least 3 independent experiments.

These findings suggested a possible AA-induced impairment in cell proliferation, or alternatively, an increase in cell death. Given that significant differences in colony size are seen as early as day 4, we examined whether AA affected early cell populations and cultured mouse embryonic stem cells under proliferating conditions for 4 days in the presence of AA. Cell proliferation-related DNA replication was monitored using the cytometric BrdU/PI assay. Although no statistically significant differences were found when comparing the percentage of BrdU positive cells following a 1 hour pulse (Figure 6A) AA showed a tendency to increase the percentage of BrdU-labelled cells (70.0 ± 1.3 % for treated cells versus 62.6 ± 4.4 % for control conditions). The small tendency to increase BrdU positive cells in the AA-treated group does not necessarily correspond to a shorter and faster cell cycle as a possible S phase lengthening would also result in augmenting the BrdU positive population. When cells pulsed with BrdU and grown for additional 4h were analyzed we found that AA-treated cells did indeed present a slower cell cycle, as the number of cells passing through S and G2/M phases and reentering G1 phase after incorporating BrdU was significantly higher in control conditions (11 ± 1.9 % in control vs 5 ± 1.2 % in AA treated cells; Figure 6B).

Cell cycle analysis provided further support for the 4 h BrdU results by showing a small but significant decrease (~ 3%) in the population in G2/M phase in AA-treated cells, and a concomitant increase in cells in S phase (~ 2%; Figure 6C). These results suggest that AA induces a slight S-phase arrest and decreases mitosis in mESC. 

Since AA is known to be a classic mitochondrial poison, we also investigated whether AA induced apoptosis and examined the activity of the executioner caspases 3 and 7 in proliferating culture conditions by performing the Caspase-glo 3/7 luminescent assay. While cells that were incubated with H_2_O_2_ (0.5 mM) for 4 hours showed significant cell death (Figure 6D), AA treatment for 4 days did not cause increased caspase-3/7 activity in mESCs when compared with control cells. These results show that the decrease in colony size observed in AA treated colonies is not due to an increased programmed cell death on mESCs populations, even though we cannot exclude the possibility that AA might have distinct effects on differentiating cells. 

### Antimycin A induces changes in adenine nucleotides

As shown in Table 1 antimycin A treatment slightly decreased adenylate energy charge (CTR = 0.87 ± 0.01 and AA = 0.83 ± 0.02, p= 0.048), with a concomitant increase in the adenylate pool (CTR = 4347.79 ± 316.45 and AA = 5372.12 ± 345.04, p= 0.038). Interestingly this increase was preferentially noticed in non-energetic adenine nucleotides (AMP) resulting in a tendency for increased AMP/ATP ratio and a significant decrease in the ATP/ADP ratios (CTR = 5.33 ± 0.53 and AA = 3.93 ± 0.41, p= 0.038). The decrease in adenylate energy charge might result from decreased energy production by oxidative phosphorylation due to AA inhibition and might contribute to the decreased proliferation observed in treated cells. 

**Table 1:** Influence of AA in adenylate energy charge, adenylate pool and adenine nucleotide ratios.

	**Energy Charge**	**Adenylate Pool** (*pmol/10* ^*6*^ *cells*)	**ATP/ADP**	**AMP/ATP**
**CTR**	0.87 ± 0.01	4347.79 ± 316.45	5.33 ± 0.53	0.07 ± 0.01
**AA**	0.83 ± 0.02*	5372.12 ± 345.04*	3.93 ± 0.41*	0.11 ± 0.03

Energy charge was calculated as ([ATP] + 0.5×[ADP])/([ATP] + [ADP] + [AMP]) and adenylate pool as ([ATP] + [ADP] + [AMP]). Statistical analysis performed by paired sample T-Student test and error = SEM. * p<0.05 vs. Control (CTR)

### Antimycin A elevates ROS levels in mESCs

**Figure 7:**
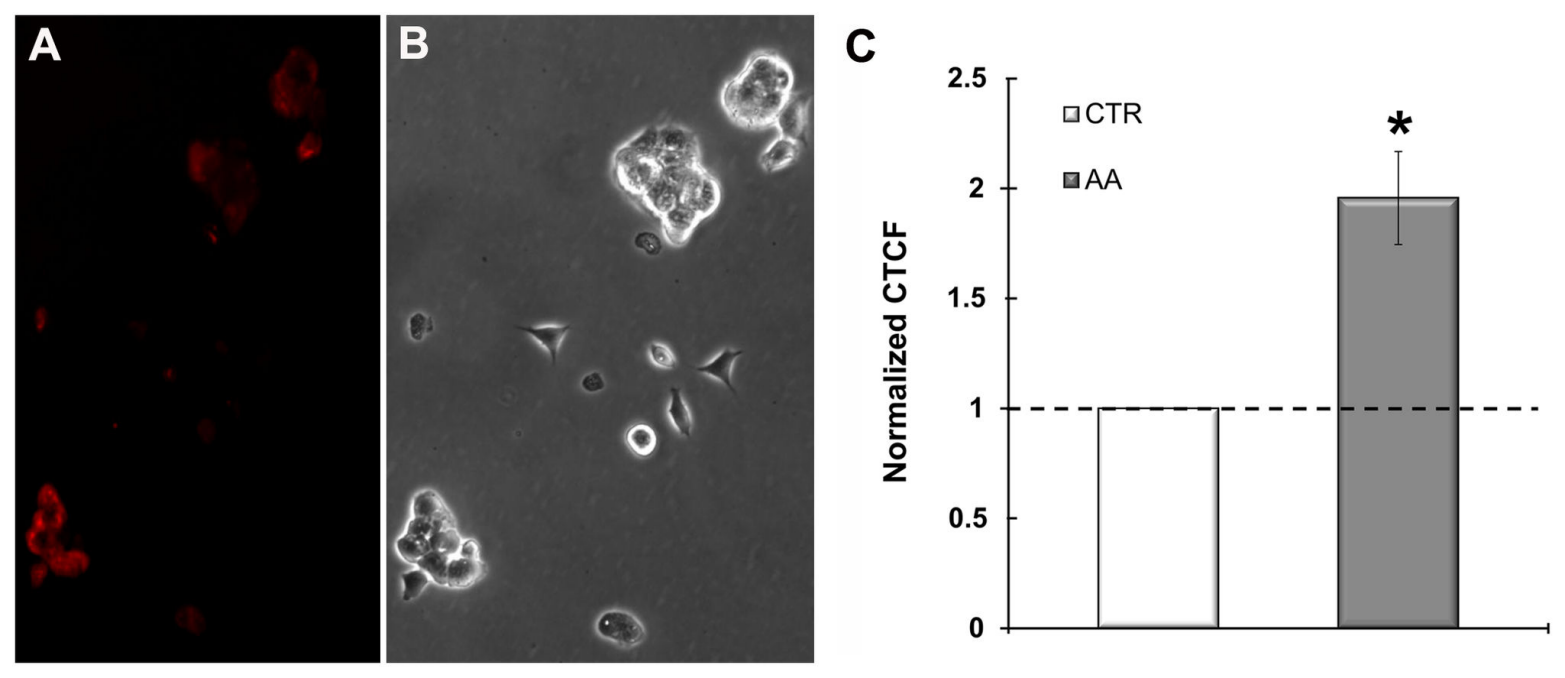
AA elevates ROS production in mESC. Superoxide anion production was evaluated through microscopic assessment of MitoSOX Red fluroscence (A). mESC incubated with 50 nM AA for 2 hours presented elevated levels of Superoxide production (red). (B). Phase contrast image of the colonies in A. (C). Fluorescence intensity was measured with ImageJ software and Corrected Total Cell Fluorescence (CTCF) was calculated through the formula (CTCF= Integrated Density – Area of Cell × Background Fluorescence) Statistical analyses were performed on raw data but for easier comprehension data were presented after normalization to the control (cells incubated with vehicle). Data were normal and homoscedastic and evaluated through paired samples T Student test. Error bars = SEM. Statistical significance considered when p≤ 0.05 (* p<0.05). Results represent 3 independent experiments.

AA is known to induce the production of Superoxide anion due to the formation and increased persistence of semiubiquinone at the Qo site of complex III [46]. Semiubiquinone is capable of univalently reducing oxygen producing Superoxide anion. In order to determine if low concentrations of AA, such as the one used in our study, induce ROS production we assessed MitoSOX Red signal in treated cells using fluorescence microscopy and image intensity quantification. As depicted in Figure 7, mESC treated for two hours with AA (50 nM) presented moderate but significantly elevated levels of Superoxide anion production when compared to the control. 

### Antimycin A elevates HIF-1α protein content in mESCs

**Figure 8:**
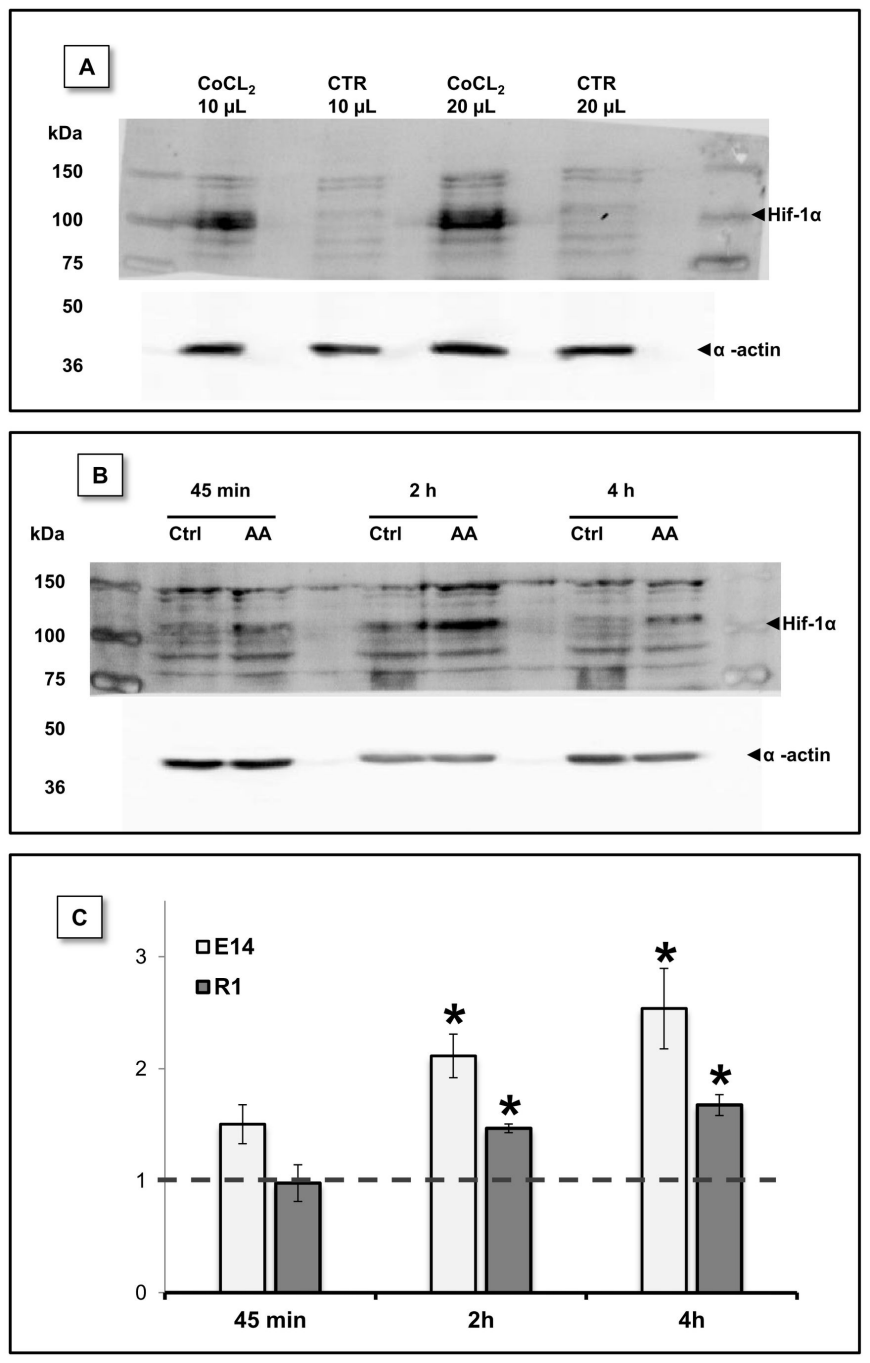
HIF-1α protein levels were stabilized by AA treatment. HIF-1α protein levels were evaluated by western blot at different time-points upon AA treatment. (A) mESC incubated for 14h with CoCl_2_ (300 µM) a known disruptor of prolyl hydroxylase activity, show increased Hif-1α protein levels. mESC without CoCl_2_ treatment were used as control and different protein volumes were loaded in the gel. (B) Representative blot for E14 cells showing increased Hif-1α protein levels downstream to AA treatment. (C) Densiometric evaluation of HIF-1α and actin (loading control) bands show that a statistical significant difference exists between control and treated conditions at 2 and 4 hours for both E14 and R1 mESC. Data shown as protein level fold increase for each one of the AA conditions relative to the respective control. Data were normal and evaluated by one sample T Student tests against a theoretical value of 1. Error bars = SEM and statistical significance is considered when * p<0.05.

Mitochondrial inhibitors have been previously shown to induce stabilization of HIF-1α, namely through the action of ROS [47,48], thus enhancing downstream pathways in distinct cellular contexts. To determine whether this protein is stabilized subsequently to AA treatment in mESCs, a western blot analysis using extracts from cells treated with AA for 45 min., 2 hours or 4 hours was performed. Figure 8 shows that HIF-1α levels were consistently elevated after AA treatment, with both cell lines depicting a significant increase when compared to the respective control for 2 and 4 hours. HIF-1α content was particularly elevated in E14 cells, increasing up to 2.54 ± 0.4 times those of the control. R1 cells treated for 4 hours showed a 1.68 ± 0.1 times protein fold increase. 

## Discussion

Many recent studies have implicated metabolic mechanisms as major regulators of pluripotent stem cells properties [49]. We have previously shown an intricate relationship between mitochondrial function modulation, as affected by antimycin A, and the maintenance of human embryonic stem cell self-renewal and pluripotency [33]. Furthermore, structural and functional remodeling of cellular metabolism has been shown to be a hallmark of various lineage specification processes, especially when the target cells have high-energy demands [26,27,29,34]. Neurons are an example of such a energy-requiring phenotype, given that a robust metabolic performance is required to support ionic pump function and thus to preserve membrane potential [34]. 

In the present work we studied the effects of mitochondrial inhibition by AA in the specific context of neuronal differentiation of mESC. We show that AA treatment abrogated mESC differentiation, as evaluated by the reduced expression of neural (Nestin) and neuronal (Tuj-1 and TH) differentiation markers and the diminished acquisition and maturation of neuronal morphology. These results are consistent with the notion that neuronal differentiation from mESC also involves a metabolic shift towards oxidative phosphorylation, with an increased cellular reliance on mitochondrial activity [28,50]. 

Previous reports have addressed the use of mitochondrial modulators (inhibitors and uncouplers) during cell differentiation processes [27,29,32,34], namely AA application has been shown to impair cardiac differentiation [27,29]. The importance of mitochondrial activity for neuronal differentiation was also proposed in earlier studies and it seems to involve much more than ATP supply. Vayssiére et al. demonstrated that neuronal-like differentiation of neuroblastoma-derived cells was hindered by the uncoupler Carbonyl cyanide 4-(trifluoromethoxy)phenylhydrazone (FCCP), but not by oligomycin (a specific inhibitor of the ATP synthase complex), implicating the existence of other MMP-related effects.

In our study we observed that initiating AA application at distinct phases of neuronal differentiation resulted in diverse outcomes. These ranged from increased cell loss, discernible by loss of colony structure for treatments initiated at later stages, to the persistence of compact pluripotent stem cell-like colonies in the cultures, observed for earlier treatment onsets (Figure 2). These observations may be interpreted as a result of the differential effect of AA in the distinct populations present in culture, which exhibit discrete metabolic maturation and thus distinct mitochondrial dependence. This metabolic diversity between distinct differentiating subpopulations was further confirmed recently [51]. Moreover, our monolayer differentiation experiments clearly showed that all neuronal lineages, and not just dopaminergic neurons, were being equally affected. This result, in conjunction with the fact that no increased cell damage was seen in those cultures after AA treatment (as evaluated by the presence of cell debris), suggested that proliferation and differentiation may be arrested at earlier neural progenitors or precursors stages. In fact, cultures treated from day 2-8 onwards presented some persistent Tuj-1^+^ cells with impaired neurite formation. Similarly, it was reported that AA treatment blocked cardiac differentiation at a precursor stage, with differentiation continuing once the AA block was removed [29]. 

AA effects during ESC differentiation seemed to involve a slight decrease in the proliferation rate, with no increased apoptosis. In contrast to what was reported for hESC [33], these results suggest that AA induces a slight arrest in S phase (with a parallel decrease of ~3% in the G2/M cell pool) which dictated a diminished proliferation rate of AA treated mESCs, as confirmed by a more than twofold increase in the G1 sub-population. Considering only the G2/M population, which includes cells that are indeed dividing at any given moment, it should be noted that a decrease of approximately 3% of total cells represents around 16% fewer cells in the population undergoing mitosis. 

Several studies have reported that interfering with mitochondrial function may lead to changes in proliferation and the cell cycle, which may be related to a decrease in ATP or modulation of ROS signaling [32,52-54]. Our data shows that AA treatment decreased adenylate energy charge with a concomitant increase in the adenylate pool (Table 1). This may account for the decreased proliferation observed due to AA, as it is known that an elevated AMP/ATP ratio can lead to the reduction of energy consuming processes (e.g. protein and lipid synthesis) some of which are needed for cell growth [55,56]. Although AA induces cell type specific responses, eliciting distinct outcomes in cell cycle modulation [52-54,57], treatment of HeLa cells with AA (10-50 µM) also resulted in a time and dose dependent S phase arrest, and even though the operating molecular mechanism was not completely elucidated, AA was capable of modulating the expression of different cyclins and the activation of proteins involved in cell cycle control such as Rb [52]. Nevertheless, we cannot completely exclude the possibility that the constraint in mESC proliferation may arise from an AA-induced elevation of ubiquinol/ubiquinone ratio, which in turn can limit pyrimidine synthesis as oxidized ubiquinone is essential for that process [58]. 

Despite the limitation detected in mESC proliferation, AA treatment conspicuously maintained Oct4 levels 4-5 times higher than the levels detected for control cultures. Simultaneously, cells in which AA was added earlier expressed lower Tuj-1 levels indicating decreased differentiation efficiency. It is worth stressing that the increment in Oct4 levels was obtained under differentiation conditions for 14 days, evidencing that AA treatment actively supported pluripotency maintenance. This reinforces the concept that mitochondria play a key role in the cellular processes involved in the choice between preserving stemness or engaging into differentiation [27,29,32,34,59,60]. As noted, some of the previous studies suggested the action of mitochondrial effects unrelated to energy status, which in the case of AA may involve mitochondrial reactive oxygen species (ROS) [33]. Controlled elevation of mitochondrial ROS is essential for the stabilization of HIF-1α protein [46,61], and here we show that incubation of mESCs with AA, resulted both in an increment in ROS levels and in the stabilization of HIF-1α under normoxia. It is likely that this signaling pathway accounts for at least some of the results obtained as hypoxia is known not only to promote a metabolic shift rendering cells more reliant on glycolysis but importantly, hypoxia inducible factors have been reported to control the expression of master regulators of stem cell pluripotency and to promote cell reprograming to a pluripotent state [49,62-64]. Therefore, the accumulation of HIF-1α in our system may encompass a metabolic conformation that will favor stem cell maintenance instead of differentiation, even under stringent differentiation conditions. 

## Conclusions

Although further work is needed in order to elucidate the molecular basis underlying these results, inhibition of mitochondrial function at the level of complex III of the ETC can prevent mESC differentiation, even under a stringent differentiation protocol, possibly by increasing ROS and HIF-1α protein levels. Modulation of mitochondrial function can therefore influence cell fate, and increasing our knowledge of the stemness/differentiation-metabolic interplay may prove an effective approach towards improving culture procedures and differentiation protocols involving pluripotent stem cells.

## Supporting Information

Figure S1 **Antimycin A hinders the formation of complex neural processes.** mESCs differentiated for 14 days using the PA6-based system were treated with AA since day 2. Control colonies presented numerous neurites forming complex structures not visible in AA-treated colonies.
(TIF)

Figure S2 **Immunocitochemistry for TuJ1 and TH markers.** E14 cells were differentiated in the absence of PA6. After fixation at day 14 of differentiation, cells were submitted to ICC for pan neuronal marker Tuj1 (green) and TH dopaminergic neuron marker (red). Control colonies display complex neuronal processes as detected by Tuj1 staining. AA treatment starting on day 2 inhibited neuronal differentiation, confirming that AA effect does not occur through feeder cells. Control conditions presented some TH positive cells that were completely absent in treated cultures.
(TIF)
