# Supplementary figures and images for: Inhibition of Mitochondrial Complex III Blocks Neuronal Differentiation and Maintains Embryonic Stem Cell Pluripotency
Source: PLoS One. 2013 Dec 2;8(12):e82095. doi: 10.1371/journal.pone.0082095 (PMC3847032; doi:10.1371/journal.pone.0082095)

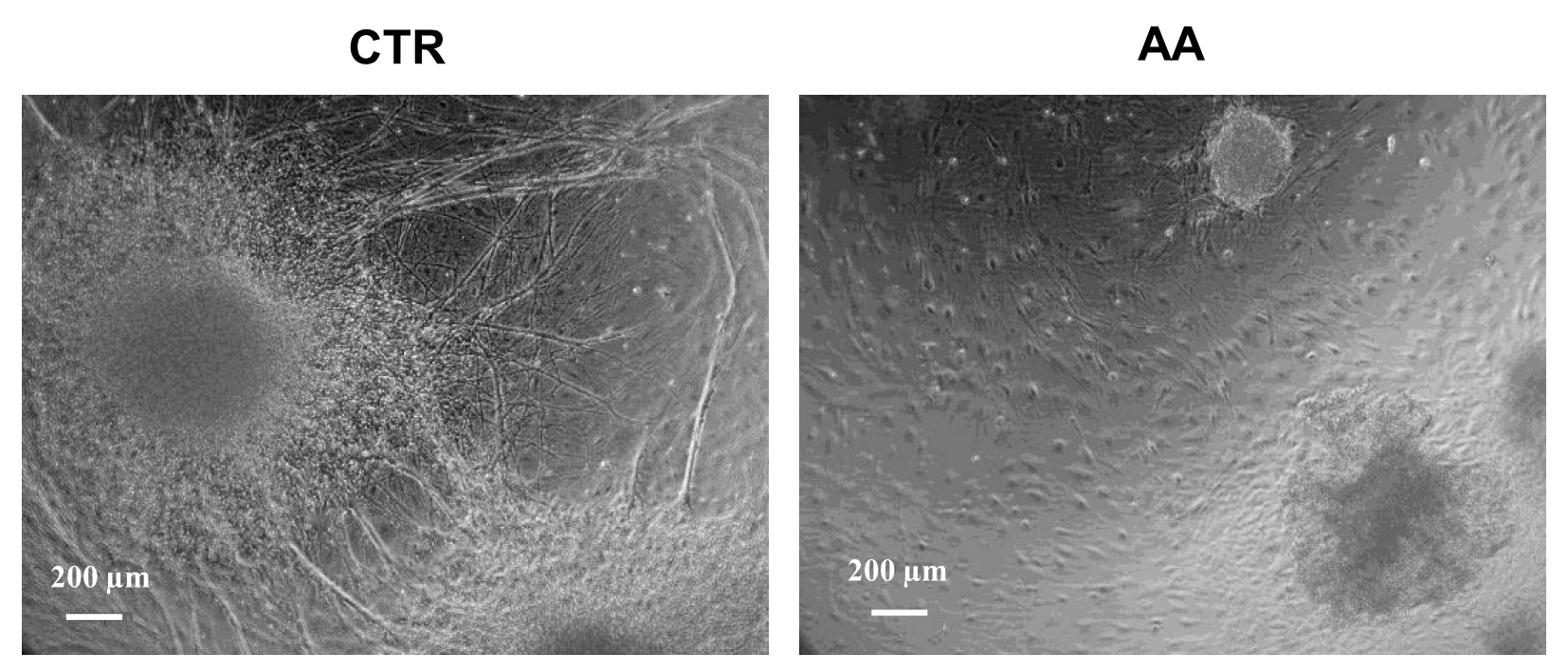

Supplement: Figure S1 — Antimycin A hinders the formation of complex neural processes. mESCs differentiated for 14 days using the PA6-based system were treated with AA since day 2. Control colonies presented numerous neurites forming complex structures not visible in AA-treated colonies. (TIF) [file pone.0082095.s001.tif]

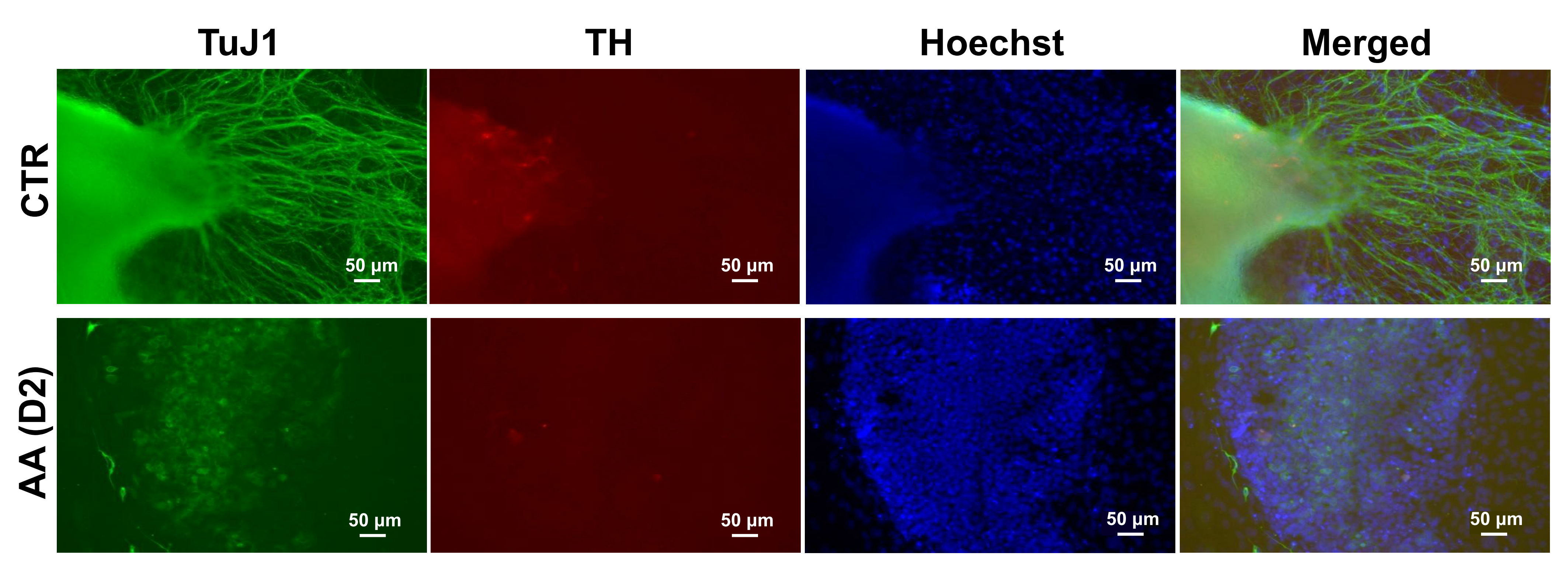

Supplement: Figure S2 — Immunocitochemistry for TuJ1 and TH markers. E14 cells were differentiated in the absence of PA6. After fixation at day 14 of differentiation, cells were submitted to ICC for pan neuronal marker Tuj1 (green) and TH dopaminergic neuron marker (red). Control colonies display complex neuronal processes as detected by Tuj1 staining. AA treatment starting on day 2 inhibited neuronal differentiation, confirming that AA effect does not occur through feeder cells. Control conditions presented some TH positive cells that were completely absent in treated cultures. (TIF) [file pone.0082095.s002.tif]
